# Needs Assessment of Perimenopause Resources and Services

**DOI:** 10.1093/geroni/igab046.2391

**Published:** 2021-12-17

**Authors:** Genna Losinski, Hilary Hicks, Pilar Thangwaritorn, Ilana Engel, Alexandra Laffer, Amy Berkley, Amber Watts

**Affiliations:** 1 University of Kansas, Lawrence, Kansas, United States; 2 University of Kansas, Kansas. City, Missouri, United States; 3 University of Kansas Medical Center, Kansas City, Kansas, United States

## Abstract

Studies show that women lack knowledge about perimenopause and feel unprepared to make healthcare decisions during this life transition. Most women want to be involved in their healthcare decisions and need timely, free, and accurate information. We conducted a needs assessment in Douglas County, Kansas by systematically reviewing regional organizations that might offer services and resources related to women’s health in midlife. We compared these resources to a benchmark for menopausal care available online nationwide (Gennev.com). We documented the primary purpose of each organization (e.g., cosmetic, wellness, medical care), services and resources offered (e.g., hormone therapy, counseling, non-pharmacological treatments), methods of outreach (e.g., blogs, classes), target audience, costs, and types of service providers (e.g., physician, counselor). We surveyed 9 regional websites: 5 offered medical care, 3 cosmetic and wellness services, 2 were municipal organizations, 2 offered mental health/social support. Four organizations offered services targeted specifically towards perimenopausal women. The most commonly offered services were hormone replacement therapy (44%), nutritional supplements (33%), and weight loss programs (33%). Very few offered educational resources (1) or menopause assessments (1) and none offered tailored psychosocial support for the perimenopause transition. The services offered were expensive with no free services and very few free resources. Organizations were primarily staffed by medical providers, only 1 organization had Menopause Practitioners certified by the North American Menopause Society. Our results demonstrate a need for comprehensive educational and support services for perimenopausal women to fulfill the need for timely, accessible, and accurate information during this understudied health transition.

